# Transcriptomic Insights into Endophytic Fungus-Mediated Enhancement of Root Growth and Stress Resistance in *Phoebe bournei*

**DOI:** 10.3390/biology15030229

**Published:** 2026-01-26

**Authors:** Zecheng Chen, Yuanyang Bi, Yuewang Niu, Jiating Chen, Cheyuan Wang, Limei You, Houhua Fu, Zongwei Zhu, Wenjun Lin, Shipin Chen, Bao Liu, Shijiang Cao

**Affiliations:** 1 Fujian Chuanzheng Communications College, Fuzhou 350007, China; 13705958911@163.com; 2 College of Forestry, Fujian Agriculture and Forestry University, Fuzhou 350002, China; yuanyangken@163.com (Y.B.); 13463995879@163.com (Y.N.); ashwcy20060601@163.com (C.W.); yolimy_y@126.com (L.Y.); fhh202216@163.com (H.F.); jaxzzw@163.com (Z.Z.); linwenjun123@126.com (W.L.); fjcsp@126.com (S.C.); fafulb@163.com (B.L.); 3 College of Art, Design and Media, Guangzhou Xinhua University, Guangzhou 510860, China; 13794567258@163.com

**Keywords:** *Phoebe bournei*, endophytic fungi, transcriptome, stress resistance, plant growth and development

## Abstract

Endophytic fungi enhance plant growth and stress resilience, yet their molecular roles in the roots of the endangered tree *Phoebe bournei* remain unclear. A comparative RNA-seq analysis was performed on root transcriptomes from wild, endophyte-colonized adult trees (OT) and axenically grown seedlings (ST). Unmapped reads were analyzed against the NCBI nucleotide (NT) database using BLASTN (v2.17.0), revealing *Rhizophagus irregularis* as the predominant endophytic fungus. Differential expression analysis identified 5891 DEGs, which were significantly enriched in pathways related to plant–pathogen interactions, phenylpropanoid biosynthesis, plant hormone signal transduction, and MAPK signaling. Key upregulated genes included *PbMPK3*, *PbCML42*, *PbCML41.2*, and *PbGSTU28*, suggesting enhanced ROS scavenging, calcium signaling, and defense activation. RT-qPCR validation confirmed the transcriptomic trends for selected genes. Our findings reveal that root endophytic fungi modulate a coordinated network involving immune priming, phytohormone regulation, and redox homeostasis, thereby supporting root development and enhancing resistance to biotic and abiotic stresses in *P. bournei*. This study provides foundational molecular insights into beneficial plant–endophyte interactions and identifies candidate genes that are valuable for the conservation and breeding of this threatened species.

## Simple Summary

Beneficial root endophytic fungi enhance growth and stress resilience in the threatened tree species *Phoebe bournei*, though their molecular mechanisms remain poorly understood. A comparison of root transcriptomes was conducted between wild trees that naturally host these fungi and seedlings cultivated under sterile conditions. Analysis of unmapped transcriptome reads identified *Rhizophagus irregularis* as the predominant symbiotic fungus. Thousands of genes exhibited altered expression, particularly in pathways related to stress defense, hormone signaling, and cellular protection. Key genes linked to root development and stress resistance, including those that regulate reactive oxygen species and calcium signaling, were validated. These findings demonstrate that beneficial fungi coordinate immune readiness, hormone balance, and cellular protection to strengthen the tree’s environmental resilience. This study establishes a foundation for the molecular breeding of this threatened species.

## 1. Introduction

Plants commonly harbor endophytic bacteria, fungi, and actinomycetes that reside asymptomatically within their tissues [1]. These endophytes boost the uptake of water and nutrients by roots and enhance resistance to biotic and abiotic stresses [2,3,4]. They colonize host plants through several mechanisms, such as cell wall modification, phytohormone modulation, effector secretion, and antioxidant production [5]. The root endophyte communities primarily arise from external microbes that infiltrate and establish themselves within roots. The efficiency of their colonization, along with the plant’s capacity to recruit these microbes, is governed by a complex regulatory network [5,6,7].

Endophytic fungi exhibit high diversity and systemically colonize plant tissues, with root-associated endophytes promoting growth and enhancing stress tolerance [8]. Under stress conditions, these fungi promote plant root growth by regulating root structure, which includes increasing root length, surface area, and branching, while also modulating various plant hormone levels [4,9]. This interaction promotes overall plant growth and enhances stress resistance. Under osmotic stress, arbuscular mycorrhizal fungi (AMF) interact with root-exuded compounds like fumarate, malate, succinate, phenolics, and proline, which also recruit beneficial microbes like Streptomyces and Gram-positive bacteria to collectively enhance plant growth [10]. Furthermore, plant endophytic fungi enhance tolerance to biotic and abiotic stresses by producing antioxidants and osmoprotectant, and modulating host pathways, thereby enabling resistance to pathogens and environmental pressures [8,9,11].

*Phoebe bournei* (Hemsl.) Yen C. Yang, an endemic Lauraceae species in China, with a straight trunk, dense evergreen canopy, and glossy leaves, is valued for landscaping, ecological restoration, and high-quality timber [12]. However, due to its high timber and economic value, *P. bournei* has long suffered from overexploitation, compounded by habitat loss from human activities and its inherently slow growth and poor natural regeneration as a tree species, collectively driving it toward endangerment [13,14]. We previously observed that cuttings of *P. bournei* exhibit poor rooting and lack root hairs (Figure S1). Root hairs significantly increase the contact area between roots and soil, thereby enhancing the efficiency of water and nutrient uptake. Plants deficient in root hairs often rely on symbiotic associations with endophytic fungi to compensate for their reduced absorptive capacity, such as orchids and blueberries [15,16]. Therefore, investigating the growth-promoting effects of root-associated microorganisms is of great significance for elite cultivar development and conservation of this species. While endophytic fungi have been shown to enhance growth and stress tolerance in multiple plant species, their specific roles and underlying molecular mechanisms in *P. bournei* remain unexplored.

To address this gap, we conducted a transcriptome comparison between roots of wild *P. bournei* and axenically cultivated seedlings. We focused on the NT alignment results of the transcriptome, which are commonly used to assess contamination and likely contain a substantial proportion of reads originating from endophytic fungi. Our aim was to identify key growth-promoting fungal taxa and to characterize the genes associated with root development and stress resistance that are modulated by these fungi, thereby enabling downstream functional validation.

## 2. Materials and Methods

### 2.1. Plant Materials and Sampling

Fine roots (<2 mm in diameter) were collected from three healthy, mature *P. bournei* trees at the Laizhou Experimental Center (26°38′2.99″ N, 118°0′18.49″ E), located in the Yangkou State-owned Forest Farm, Fujian Province. The field-collected roots designated OT (endophyte-colonized) were rinsed with sterile water, cut into approximately 2 cm segments, pooled, flash-frozen in liquid nitrogen, and stored at −80 °C. In comparison, roots from axenically grown seedlings were labeled ST (sterile control) and processed identically.

### 2.2. Endophytic Fungal Staining and Paraffin Section Observation

Root endophyte staining was performed following Phillips & Hayman with minor modifications [17]. Roots were fixed in FAA (5% formalin: 5% acetic acid: 90% ethanol) at 4 °C for 24 h, rinsed, and cleared in 100 g L^−1^ KOH at 90 °C for 90–120 min. After three water washes, samples were bleached with 30% (*w*/*w*) H_2_O_2_ for 5 min, rinsed again, and acidified in lactic acid for 5 min. They were then stained with 5% ink–vinegar for 3–5 min, rinsed, and soaked overnight in water. For observation, thick roots were split underwater to remove the central vascular cylinder, teased into thin sheets, mounted on slides, and sealed with clear nail polish. Paraffin sectioning was performed as described by Miya [18].

### 2.3. RNA Sequencing and Analysis

Total RNA was isolated from root tissues of field-grown mature trees and axenically grown seedlings using the YALEPIC^®^ Plant Total RNAFast Isolation Kit (PLUS) (Yali Biotech, Suzhou, China). RNA samples were then submitted to BGI Genomics (Shenzhen, China) for mRNA transcriptome sequencing. The raw data were processed using fastp (v 0.23.2) [19] and FastQC (v 0.11.9) [20] for quality filtering and control to obtain clean reads. Clean reads were mapped to the chromosome-scale genome of *P. bournei* using STAR (v2.7.10a) [21]. This genome assembly CNSA (accession CNP0002030) was reported by Han [12] (https://ftp.cngb.org/pub/CNSA/data5/CNP0002030/CNS0395682/CNA0029376/ (accessed on 15 October 2025)). To detect potential microbial contamination, unmapped reads were aligned against the NCBI NT database using BLASTN (v2.17.0) [22]. RSEM (v1.3.1) quantified expression [23]. DESeq2 (v1.34.0) identified DEGs (q < 0.05 and |log_2_FC| > 1) [24]. ClusterProfiler (v4.2.2) performed functional enrichment [25].

### 2.4. PPI Network Analysis

Protein sequences from DEGs enriched in GO terms and KEGG pathways were submitted to the STRING database for the construction of two independent PPI networks (https://cn.string-db.org (accessed on 10 December 2025)). These networks were exported and visualized using Cytoscape (v3.10.1) for refinement [26]. *Arabidopsis thaliana* was selected as the reference organism, and the minimum required interaction confidence score was set to 0.7 (high confidence). All other parameters were kept at their default settings.

### 2.5. Expression Pattern Analysis of Genes in Key Functional Modules

Based on the PPI network analysis, key genes were selected for expression pattern analysis. Heatmaps were generated using TBtools (v2.386) [27], and final graphical refinement was performed with Adobe Illustrator (v2020).

### 2.6. Quantitative Real-Time PCR (RT-qPCR) Analysis

First-strand cDNA synthesis was performed using ABKscript RT MasterMix with gDNA Remover (AiBiKang Biotechnology, Xiamen, China). Detailed information on the reaction composition and thermal cycling parameters is detailed in Supplementary Table S1. RT-qPCR was performed using ABKfast Universal qPCR Mix (SYBR Green I chemistry; AiBiKang Biotechnology, Xiamen, China). The full reaction setup and amplification program are listed in Supplementary Tables S2 and S3. Gene expression was normalized to *PbEF1α* using the 2^−ΔΔCT^ method [28], based on four biological replicates. Primers for *PbEF1α* (GeneBank No. KX682032) amplification were 5′-CATTCAAGTATGCGTGGGT-3′ and 5′-ACGGTGACCAGGAGCA-3′ [29]. Primer sequences for all other target genes are provided in Supplementary Table S4.

## 3. Results

### 3.1. Transcriptome Data Processing

To elucidate the molecular mechanism underlying endophyte-mediated root development in *P. bournei*, we performed transcriptome sequencing of roots from field-collected wild plants and axenically cultured seedlings. Each sample yielded an average of 42.2 million clean reads, with 75.2–92.9% aligning to the *P. bournei* reference genome (Table S5), thereby ensuring adequate coverage for subsequent expression analysis. The mapping rates of OT samples (75.17–87.54%) were significantly lower than those of ST samples (92.50–92.90%), indicating the presence of endophyte-derived sequences in the OT transcriptomes. To ascertain the potential biological sources of unmapped reads, we conducted a BLAST-based alignment of these reads from OT and ST group samples against the NCBI nt database. Notably, *Rhizophagus irregularis*, a known AMF, was identified in the unmapped reads of OT samples, providing direct evidence for the presence of endophytic fungi in field-collected plants (Table S6).

### 3.2. Differential Gene Expression and Functional Enrichment Analysis

**Figure 1:**
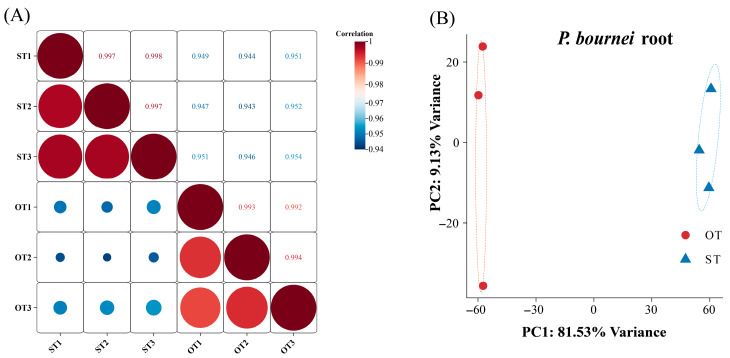
Sample relationships between OT and ST groups. (**A**) Pearson correlation heatmap of gene expression; (**B**) PCA plot (PC1 vs. PC2). OT denotes field-collected wild plants, and ST denotes axenically cultured seedlings. The numbers 1–3 indicate three biological replicates for each group.

Sample correlation analysis was conducted within the OT and ST groups to evaluate biological reproducibility and intergroup divergence. Samples within each group showed high expression correlation (large, red circles), while intergroup correlations were low (small, blue circles) (Figure 1A). PCA distinguished OT and ST samples into two distinct clusters, with PC1/PC2 capturing 90.66% of the total variance (81.53%/9.13%) (Figure 1B), confirming robust within-group reproducibility for subsequent analyses.

**Figure 2:**
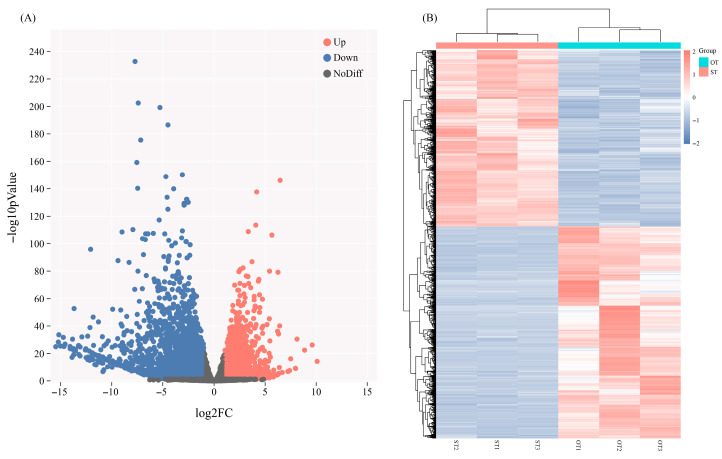
Identification and clustering of DEGs. (**A**) Volcano plot of DEGs; (**B**) Heatmap of DEGs.

To examine the influence of endophytic fungi on *P. bournei* root physiology, we compared OT and ST transcriptomes, identifying 5891 DEGs (|log_2_FC| > 1, FDR < 0.05), comprising 3220 up- and 2671 down-regulated genes (Figure 2A,B).

**Figure 3:**
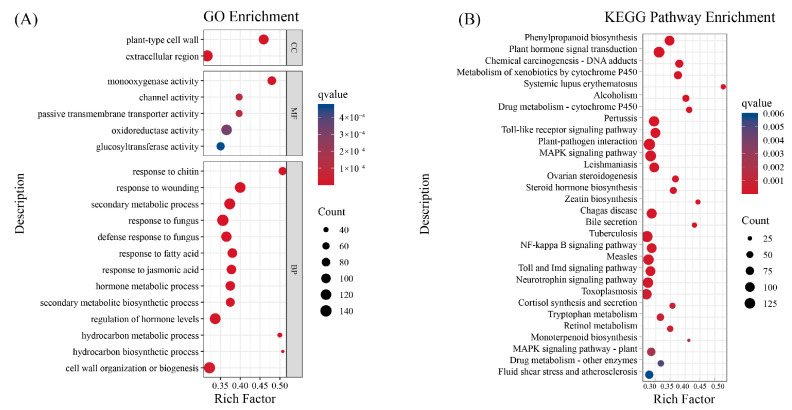
Enrichment analysis of DEGs. (**A**) GO enrichment analysis; (**B**) KEGG pathway enrichment analysis.

GO and KEGG pathway enrichment analyses were conducted and the top 20 most significantly enriched terms were visualized according to q-value. GO analysis demonstrated enrichment of DEGs in plant-type cell wall and extracellular region (CC, cellular component), monooxygenase/oxidoreductase/glucosyltransferase activity (MF, molecular function), and biological processes (BP) including response to chitin, wounding, fungus, jasmonic acid, and secondary metabolism, suggesting roles in cell wall remodeling, redox regulation, and defense activation (Figure 3A). KEGG enrichment identified phenylpropanoid biosynthesis, plant hormone signal transduction, plant–pathogen interaction, and MAPK signaling as significantly enriched pathways, suggesting that endophytic fungi may promote growth via immunity, signaling, and secondary metabolism regulation (Figure 3B).

### 3.3. Protein–Protein Interaction (PPI) Network of Key Pathways

**Figure 4:**
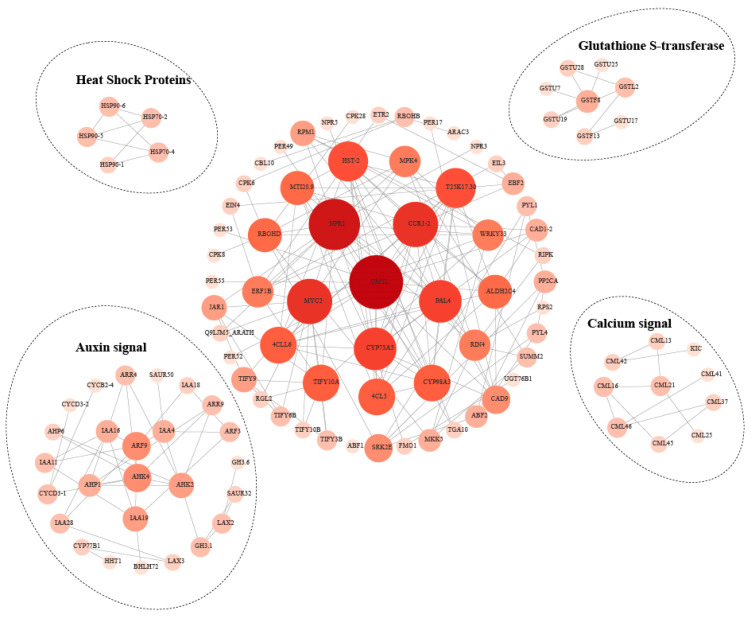
PPI network of DEGs enriched in key KEGG pathways. Nodes represent proteins; Edges represent interactions. Node size and color intensity reflect centrality: higher centrality yields larger and darker nodes.

Using *A. thaliana* as a reference, PPI networks were constructed separately for key enriched GO terms and KEGG pathways. The PPI network of DEGs from key GO terms was clustered into six functional modules (Figure S2). The central hub included core regulatory proteins such as MYC2, NPR1, WRKY33, PAL4, MPK3, and CYP74A, while the other five modules were associated with gibberellin and cytokinin signal, jasmonate signal, lignin biosynthesis, Brassinosteroid signal, and auxin biosynthesis and signaling (Figure S2). The PPI network of DEGs from key KEGG pathways was clustered into five functional modules (Figure 4). The central hub contained hub proteins such as OMT2, CCR1-2, and CYP73A5, while MYC2, NPR1, PAL4, and WRKY33—previously identified in the GO-based network—were also prominent here, indicating their pivotal roles in endophytic fungus-mediated root development. The surrounding modules were enriched in heat shock response, glutathione S-transferase activity, calcium signaling, and auxin signaling. Collectively, these hub proteins may serve as molecular switches coordinating plant growth and stress adaptation in response to endophytic colonization.

### 3.4. Analysis of Endophytic Fungus-Regulated Genes

**Figure 5:**
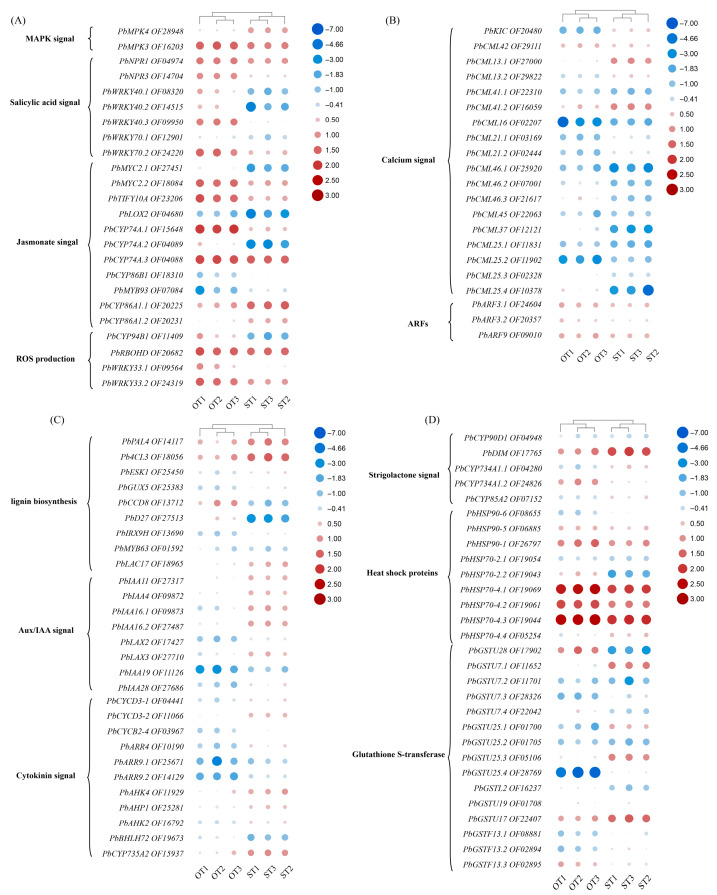
Expression heatmap of key PPI network genes, categorized by putative function. (**A**) Genes related to MAPK signal, Salicylic acid signal, Jasmonate singal and ROS production. (**B**) Genes related to Calcium signal and ARFs. (**C**) Genes related to lignin biosynthesis, Aux/IAA signal and Cytokinin signal. (**D**) Genes related to Strigolactone signal, Heat shock proteins and Glutathione S-transferase. Red represents high expression, blue low; the size of the dots and the intensity of their color both increase with expression level.

Based on the PPI network, we selected hub genes from key modules and analyzed their expression patterns to infer potential physiological functions. The MPK3/MPK6 and MPK4 cascades exhibit deeply conserved MAPK signaling pathways across eukaryotes, which are crucial for coordinating plant development and immunity [30]. *PbMAPK3* and *PbMAPK4* were detected in both OT and ST samples, with *PbMAPK3* exhibiting significantly higher expression in OT than in ST (Figure 5A). These results suggest that the *MPK3/MPK6* cascade may mediate endophyte-mediated regulation of root development in *P. bournei*. Additionally, genes related to the salicylic acid signaling pathway, such as *PbNPR1*, *PbNPR3*, and *PbWRKY40.3*, were significantly upregulated in OT samples compared to ST samples. Some plant growth-promoting rhizobacteria (PGRB) produce jasmonic acid to modulate immunity and induce systemic resistance. As a core JA signaling transcription factor, *MYC2* activates root defense responses. In our data, *PbMYC2.1* and *PbMYC2.2* were significantly upregulated in OT versus ST roots, while the JA repressor *TIFY10A* was also highly expressed in OT, suggesting complex JA pathway modulation by endophyte [31]. In the ROS (reactive oxygen species) related genes, endophytic fungi activated genes mediating both ROS generation and scavenging. The ROS producing gene *PbRBOHD* and antioxidant/defense-associated genes (*PbCYP94B1*, *PbWRKY33.1*, and *PbWRKY33.2*) were all significantly upregulated.

Calcium signaling regulates developmental and stress responses in plants [32]. In the calcium signaling category, the calcium-sensing gene *PbCML42* was induced during root–endophyte interactions, suggesting its potential involvement in endophytic colonization and growth promotion in roots (Figure 5B). Auxin response factors (ARFs) were significantly upregulated in the OT group, suggesting that endophytic fungi may promote root development by inducing auxin signaling.

Among genes associated with lignin biosynthesis, most were significantly upregulated in the ST group, whereas *PbMYB63* exhibited higher expression in the OT group (Figure 5C). This suggests that lignin biosynthesis is more active in ST roots than in OT roots. Aux/IAA signaling pathway genes were significantly upregulated in the ST group but maintained at moderate levels in the OT group. Similarly, cytokinin signaling–related and strigolactone signal genes showed a comparable expression pattern, likely because the ST roots are derived from seedlings undergoing active growth (Figure 5C,D). Heat shock proteins were significantly upregulated in the OT group, suggesting that endophytic fungi may enhance the plant’s defense capacity against abiotic stress. Within the glutathione S-transferase (GST) gene cluster, *PbGSTU28* was markedly activated. Additionally, plant catalases are key enzymes responsible for hydrogen peroxide degradation, while glutathione S-transferases participate in oxidative stress scavenging [33]. Beyond its roles in maintaining cellular redox homeostasis and detoxification, glutathione also serves as a critical hub that integrates signaling networks and metabolic processes to facilitate plant growth and development [34].

### 3.5. Validation of Key Gene Expression by RT-qPCR

**Figure 6:**
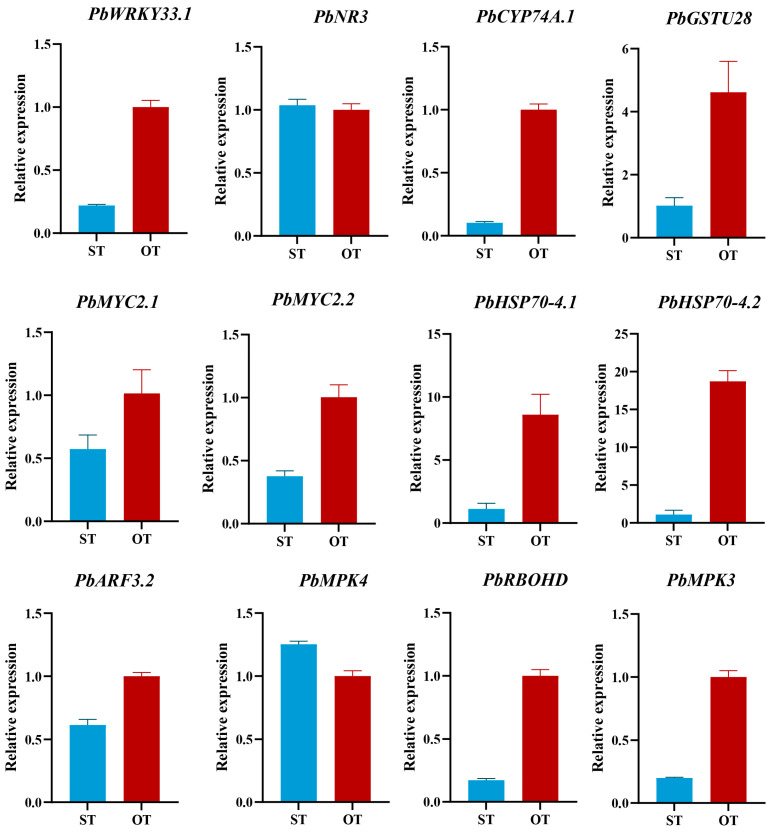
Relative expression levels of key genes.

Based on the PPI network and heatmap, key genes associated with endophyte-mediated enhancement of root defense and growth were validated by real-time quantitative PCR (RT-qPCR). Expression profiles of these genes matched the transcriptomic trends, confirming the reliability of the RNA-seq results (Figure 6).

## 4. Discussion

Endophytic fungi enhance plant growth, nutrient acquisition and stress resistance through symbiotic interactions [35,36,37,38]. For instance, AMF can improve plant growth and nutritional conditions by increasing soil nutrient availability, including phosphates and nitrates, while also bolstering plant resistance to stressors such as drought and heavy metal toxicity [39]. Furthermore, inoculating ericoid mycorrhizal fungi (EMF) Oidiodendron maius strain BL01 into blueberries enhanced the absorption efficiency of ammonium nitrogen [16]. Preliminary observations revealed that *P. bournei* lacks root hairs (Figure S1). Wild plants harbor endophytic fungi in their roots, whereas axenically grown seedlings show slightly reduced growth, root diameter, and root biomass. Therefore, it is hypothesized that endophytic fungi may promote the formation and development of lateral roots, enhancing the roots’ capacity for nutrient and water acquisition, thereby facilitating overall plant growth.

### 4.1. Immune Priming and Fine-Tuned Defense Responses

We compared root transcriptomes of field-grown mature *P. bournei* (OT, naturally colonized by fungi) and axenically grown seedlings (ST). During alignment of OT samples to the *P. bournei* reference genome, we observed a relatively lower mapping rate in the OT group compared to the ST group. Since our sampling process did not involve external contamination, we speculate that some of the unmapped reads in OT may originate from endophytic fungi. Unmapped reads alignment confirmed our hypothesis, with *R. irregularis* ranking first in OT group. Subsequently, we performed endophytic fungal staining, which directly confirmed this observation (Figure S3). This species is a widely distributed and well-studied AMF that forms obligate symbiosis with approximately 80% of terrestrial plants [40]. PRRs detect fungal MAMPs such as chitin via the cell wall, initiating immune responses that can also facilitate beneficial symbiosis following endophytic colonization [41]. GO terms such as response to wounding, response to fungus, and defense response to fungus were significantly enriched, along with the KEGG pathway plant–pathogen interaction, further suggesting that the root cell wall of *P. bournei* possesses a fine-tuned capacity to distinguish beneficial endophytic fungi, enabling selective colonization while maintaining defense against potential pathogens. A similar enrichment of plant-type cell wall organization was reported in Arabidopsis colonized by *Aureobasidium* sp. JRF1 [38], suggesting these pathways may positively regulate endophyte recruitment and, through the activation of immune responses, enhance resistance against pathogenic fungi. The plant–pathogen interaction pathway is a complex regulatory network that reflects both the activation of defense responses and accommodation mechanisms toward endophytes in plants. Genes associated with the MAPK signaling cascade, particularly the MAPKKK3/5–MKK4/5–MPK3/6 kinase module, mediates the regulation of plant growth and disease resistance [42]. In this study, two MPK genes were significantly upregulated in OT, especially *PbMPK3*, suggesting that the *PbMPK3*-mediated signaling cascade enhances root resistance to both biotic and abiotic stresses.

### 4.2. Modulation of Phytohormone Networks for Growth and Colonization

Endophytic fungi can modulate endogenous phytohormone levels—such as jasmonic acid, auxin, and cytokinin—to promote root development and overall plant growth [43,44]. Moreover, previous studies have shown that inoculation with strains of *Aureobasidium* sp. upregulates auxin- and cytokinin-related genes while suppressing the jasmonic acid/ethylene signaling pathway, thereby promoting plant growth [38]. In GO terms, response to jasmonic acid, hormone metabolic process, and regulation of hormone levels were extremely enriched. In KEGG pathways, plant hormone signal transduction, zeatin biosynthesis, as well as steroid hormone biosynthesis were also markedly enriched. These results indicate that endophytic fungi can indeed modulate endogenous phytohormone levels, thereby influencing root development and overall plant growth. In this research, *ARF* genes were predominately upregulated in the OT group, along with *PbMYC2.1* and *PbMYC2.2*—key transcriptional regulators of salicylic acid signaling—also showing elevated expression in OT roots. This pattern underscores the growth-promoting role of endophytic fungi in root development. Cytokinins are plant hormones that regulate cell division and differentiation. In this study, cytokinin signaling–related genes showed moderate expression in OT, demonstrating that cell division in the root apical meristem may proceed at a relatively slower rate—potentially facilitating endophytic fungal colonization. Plant roots secrete strigolactones into the rhizosphere, and the compounds serve as key symbiotic signaling molecules that can stimulate fungal spore germination and hyphal branching, thereby initiating the colonization process. Two strigolactone-related genes, *PbCYP734A1.2* and *PbDIM*, were significantly expressed in the OT group and may participate in this process.

### 4.3. Enhanced Redox Homeostasis and Secondary Metabolism Underpin Stress Adaptation

Genes in the phenylpropanoid pathway help produce diverse metabolites; for example, class III peroxidases in *Arundo donax* enhance stress tolerance by boosting secondary metabolite accumulation [45]. ROS act as signaling mediators that regulate phenylpropanoid biosynthesis, and genes associated with ROS production were significantly upregulated in the OT group. It is speculated that endophytic fungi may stimulate genes involved in ROS production such as *PbRBOHD*—thereby inducing phenylpropanoid pathway genes to produce antioxidant metabolites that modulate plant growth, stress responses, and antioxidant defense. Glutathione S-transferases alleviate oxidative stress and interact with calcium signaling pathways, thereby influencing signal transduction, transcription factor activity, and apoptosis [46]. In this study, *PbGSTU28* and *PbGSTSU17* were significantly upregulated in OT1, suggesting they help maintain cellular redox homeostasis and detoxification functions, thereby supporting plant growth. Calcium signaling coordinates both xylem differentiation and plant stress responses [47]. In this study, calcium signaling genes *PbCML42* and *PbCML41.2* were significantly upregulated in the OT group, suggesting that they may adjust the expression of genes which are contained in the lignin biosynthesis as well as the heat shock proteins in order to promote plant growth and enhance defense against abiotic stresses.

### 4.4. A Coordinated Signaling Cascade from Perception to Output

Collectively, our findings support a model in which endophytic fungi regulate key gene expression in *P. bournei* through a multi-layered signaling cascade. Firstly, fungal MAMPs activate host PRRs, triggering MAPK cascades and calcium signaling to initiate early immune responses, as evidenced by the upregulation of *PbMPK3* and *PbCML42*. Secondly, the induced calcium signals and ROS signals jointly reprogram hormone networks, establishing a JA/auxin-dominated and SA-suppressed pattern, which coordinates growth and defense priorities. This is reflected in the significant upregulation of PbMYC2.1/2.2 and ARF genes alongside the modulation of SA-related genes. Thirdly, downstream transcription factors (e.g., *MYB63*, *WRKYs*) likely redirect metabolic flux from lignin synthesis toward the biosynthesis of antioxidant and anti-microbial compounds (e.g., phenylpropanoids), directly enhancing stress tolerance. This forms a complete “perception–integration–output” signaling cascade, explaining the symbiosis-induced synergistic enhancement of growth and stress resistance observed in *P. bournei*.

## 5. Conclusions

This study demonstrates that root endophytic fungi enhance growth and stress tolerance in *P*. *bournei* through coordinated regulation of immune responses, phytohormone signaling, and redox homeostasis. Analysis of unmapped transcriptomic reads revealed significant enrichment of *R*. *irregularis*, a widespread arbuscular mycorrhizal fungus known to foster plant development and stress resistance. Endophyte colonization led to the upregulation of key genes involved in MAPK signaling (*PbMPK3*), calcium signaling (*PbCML42* and *PbCML41.2*), ROS scavenging (*PbGSTU28*), lignin biosynthesis, and phenylpropanoid metabolism. Together, these transcriptional changes support root development while priming plant defense against both biotic and abiotic stresses. Our findings provide valuable molecular insights into beneficial plant–endophyte interactions and identify promising candidate genes to support the conservation and breeding of this endangered tree.

## Data Availability

Statistical analyses are included in the Supplementary Materials. All other data are available from the corresponding author upon reasonable request. Further enquiries can be directed to the corresponding author.

## Supplementary Materials

The following supporting information can be downloaded at https://www.mdpi.com/article/10.3390/biology15030229/s1, Figure S1: Root morphology and paraffin section of *P. bournei*; Figure S2: PPI network of DEGs enriched in key GO terms; Figure S3: Endophytic fungi in the root system in *P. bournei*; Table S1: Composition of the reverse transcription reaction mixture; Table S2: Composition of the qRT-PCR reaction mixture; Table S3: qRT-PCR amplification programs; Table S4: Primer sequences used in this study; Table S5: Reads quality statistics and comparison results of reference genome; Table S6: Taxonomic classification of unmapped reads using nucleotide (nt) reference database.

## Abbreviations

The following abbreviations are used in this manuscript:

AMF	arbuscular mycorrhizal fungi
BP	Biological processes
CC	Cellular component
CNSA	China National GeneBank DataBase
DEGs	Differentially expressed genes
EMF	Ericoid Mycorrhizal Fungi
FAA	Formalin–acetic acid–alcohol
GO	Gene Ontology
KEGG	Kyoto Encyclopedia of Genes and Genomes
MF	Molecular function
MAMPs	Microbe-Associated Molecular Patterns
OT	wild, endophyte-colonized adult trees
PRRs	Plant pattern recognition receptors
ROS	Reactive oxygen species
RT-qPCR	Real-time quantitative polymerase chain reaction
ST	axenically grown seedlings
