# Supplementary material for: Transcriptomic Insights into Endophytic Fungus-Mediated Enhancement of Root Growth and Stress Resistance in Phoebe bournei
Source: Biology (Basel). 2026 Jan 26;15(3):229. doi: 10.3390/biology15030229 (PMC12896463; doi:10.3390/biology15030229)
Supplement: Supplementary file 1 [file biology-15-00229-s001.zip › Supplementary Figures.pdf]

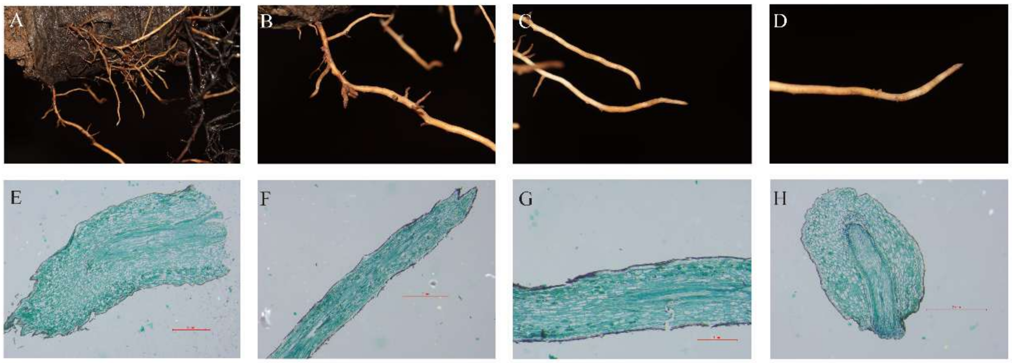

FigureS1. Root morphology and paraffin section of *P. bournei*. A-D: Root morphology; E-H: Paraffin section.

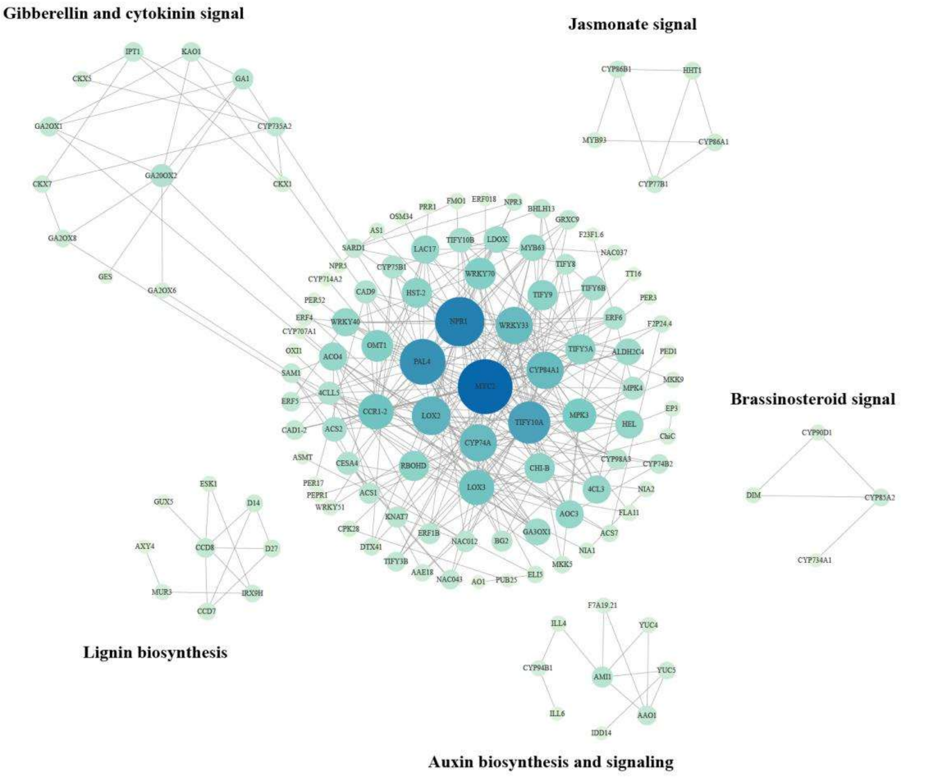

FigureS2. Protein–protein interaction (PPI) network of DEGs enriched in key Go terms. Each node represents a protein, and edges indicate interactions. Node size and color intensity are scaled according to the protein's centrality, with larger and darker nodes occupying more central positions in the network.

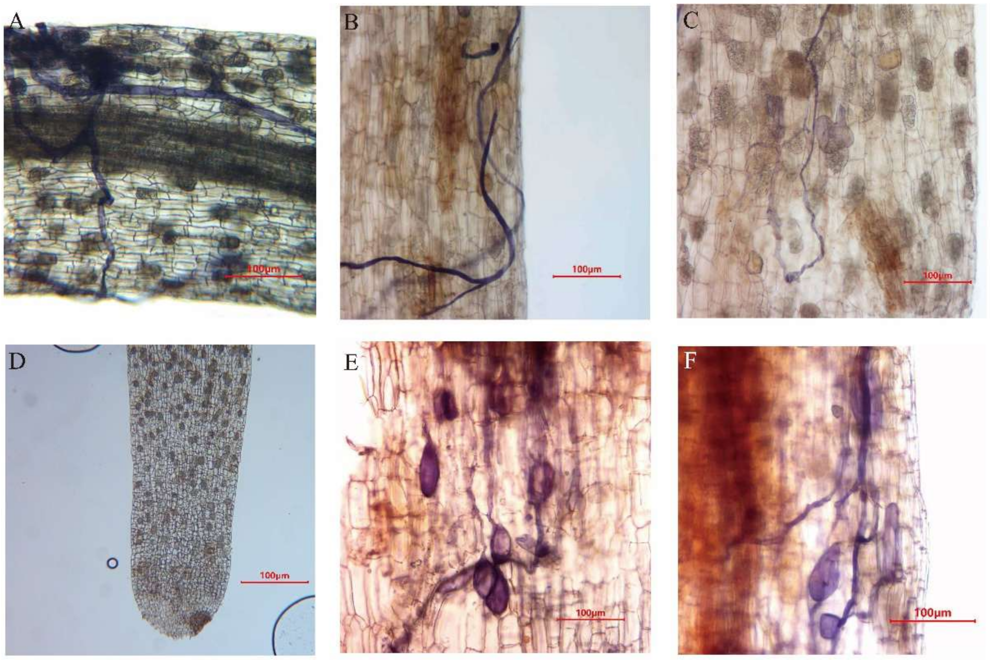

FigureS3. Endophytic fungi in the root system in *P. bournei*. A-C: Hyphae and hyphal aggregates; D-F: Hyphae and vesicular structures.
